# Typical gray matter axons in mammalian brain fail to conduct action potentials faithfully at fever‐like temperatures

**DOI:** 10.14814/phy2.12981

**Published:** 2016-10-05

**Authors:** Dobromila Pekala, Hanna Szkudlarek, Morten Raastad

**Affiliations:** ^1^ Department of PhysiologyEmory University School of MedicineAtlantaGeorgia; ^2^ Department of Anatomy & Cell BiologyThe Schulich School of MedicineUniversity of Western OntarioLondonOntarioCanada

**Keywords:** Conduction failures, excitability, gray matter axons, presynaptic mechanisms

## Abstract

We studied the ability of typical unmyelinated cortical axons to conduct action potentials at fever‐like temperatures because fever often gives CNS symptoms. We investigated such axons in cerebellar and hippocampal slices from 10 to 25 days old rats at temperatures between 30 and 43°C. By recording with two electrodes along axonal pathways, we confirmed that the axons were able to initiate action potentials, but at temperatures >39°C, the propagation of the action potentials to a more distal recording site was reduced. This temperature‐sensitive conduction may be specific for the very thin unmyelinated axons because similar recordings from myelinated CNS axons did not show conduction failures. We found that the conduction fidelity improved with 1 mmol/L TEA in the bath, probably due to block of voltage‐sensitive potassium channels responsible for the fast repolarization of action potentials. Furthermore, by recording electrically activated antidromic action potentials from the soma of cerebellar granule cells, we showed that the axons failed less if they were triggered 10–30 msec after another action potential. This was because individual action potentials were followed by a depolarizing after‐potential, of constant amplitude and shape, which facilitated conduction of the following action potentials. The temperature‐sensitive conduction failures above, but not below, normal body temperature, and the failure‐reducing effect of the spike's depolarizing after‐potential, are two intrinsic mechanisms in normal gray matter axons that may help us understand how the hyperthermic brain functions.

## Introduction

Most central and peripheral mammalian axons conduct action potentials (spikes) with great fidelity at normal body temperature and down to 5–15°C (Franz and Iggo 1968), but above physiological temperature, peripheral axons sometimes fail. Temperature‐induced conduction failures have been studied for more than 100 years (Bernstein 1902). They are known to occur in several nonmammalian preparations (Swadlow et al. 1980), and also in humans with demyelinating disease (Rasminsky 1973; Howells et al. 2013), but whether conduction failures occur in normal axons in the mammalian brain at elevated temperatures as seen during fever is not known.

Theoretically, conduction failures could contribute to the drowsiness we experience during fever, and to clinical syndromes like “febrile seizures”, which is the most common pathological brain activity in young children (Dube et al. 2007). The identification of temperature‐sensitive conduction failures in mammalian brain may therefore contribute to a more mechanistic understanding of such symptoms and pathology.

The very thin unmyelinated axons in cortex and many other gray matter areas may face greater challenges than other thicker or myelinated axons at elevated temperatures because of their very high axial electrical resistance, very large surface‐to‐volume ratio, extensive branching, and variable diameter along their paths. Particularly, variations in diameter and branching (Swadlow et al. 1980) reduce conduction reliability by creating regions of “impedance mismatch”, for example, in squid (Westerfield et al. 1978), leech (Yau 1976), and vertebrate sensory ganglia (Dun 1955). High temperature in combination with such impedance mismatch is known to elicit failures in some branching axons, for example, in the squid (Westerfield et al. 1978) and vertebrate sensory axons (Barron and Matthews 1935).

The mechanism by which high temperature elicits conduction failures is probably by reducing the width and amplitude of the spike (Hodgkin and Katz 1949). Conversely, factors that increase spike amplitude and/or duration, for example, pharmacological block of spike repolarizing K^+^ currents, are expected to reduce conduction failures (Bostock et al. 1978; Westerfield et al. 1978). In the cerebellar parallel fibers, spike broadening has been recorded with voltage‐sensitive dyes in response to 1 mmol/L TEA (Sabatini and Regehr 1997). We will utilize this relatively well‐established pharmacology of the parallel fiber spike's repolarization to test if conduction failures, as expected, are reduced by spike broadening.

Furthermore, if failures are due to geometric factors combined with a temperature‐induced reduction of spike amplitude and/or width, one may expect moderate depolarization of the axonal membrane to reduce failures, at least in a temperature range where the spikes are not severely attenuated. Interestingly, typical cortical thin axons and several peripheral thin axons have an intrinsic mechanism, the depolarizing after‐potential (DAP), that increases axonal excitability for 50–100 msec after a spike (Gardner‐Medwin 1972; Wigstrom and Gustafsson 1981; Palani et al. 2012), and may therefore reduce conduction failures.

To identify conduction failures, the spikes need to be recorded at least at two positions along the axons, one close to the stimulation site to confirm that spikes are initiated, and the other more distal to check if spikes arrive further out in the axon. The reason to distinguish initiation failures and conduction failures is that those two types of failures probably have very different causes and consequences (Swadlow et al. 1980). Initiation of spikes, which can be temperature sensitive (Takeya et al. 2002), usually takes place in the axon's initial segment and is a regulated, homeostatic process (Adachi et al. 2015). Conduction failures on the other hand are probably not under the same control and may be caused by a wide range of normal or pathological factors, including axonal structure and nonuniform distribution of membrane currents. Also, the network effect will be different if the whole axon fails, compared to if it fails at a specific point far from the soma.

Although two recording sites on individual typical unmyelinated, cortical axons have not been achievable, it is possible to record from bundles of them at different distances from the stimulation site. If higher temperature gives conduction failures between the proximal and distal electrode, it may be detected as a preferential drop in the population response at the distal recording site. For example, the cerebellar parallel fibers showed such distal‐recording drop at high frequencies when spikes had to pass the branch point, but not when spikes were recorded at two sites after the branch point (Baginskas et al. 2009).

Despite their structural predispositions for conduction failures, typical unmyelinated cortical axons, like cerebellar parallel fibers and axons in hippocampal stratum radiatum, conduct spikes reliably at normal body temperature (Eccles et al. 1966; Frenguelli and Malinow 1996; Mackenzie et al. 1996; Cox et al. 2000; Forti et al. 2000; Koester and Sakmann 2000; Raastad and Shepherd 2003; Brenowitz and Regehr 2007). An important remaining question is therefore if temperatures above normal body temperature could elicit conduction failures in such axons.

## Materials and Methods

Experiments were conducted according to the National Institutes of Health Guidelines for Animal Research. All animals were treated in accordance with the regulations of the Institutional Animal Care and Use Committee at Emory University, GA. We obtained 177 successful recordings from 95 rats.

### Slice preparation

Wistar and Sprague–Dawley rats of either sex (10–25 days old) were used. Slices of cerebellum and hippocampus were prepared as described previously (Pekala et al. 2014). Briefly, the animal was exposed to isoflurane and decapitated after loss of consciousness and pain reflexes.

The brain was quickly removed and submerged in oxygenated (5% carbogen), ice‐cold artificial cerebrospinal fluid (ACSF, in mmol/L): glycerol 250, KCl 1.25, KH_2_PO_4_ 1.25, MgCl_2_ 7, NaHCO_3_ 25, CaCl_2_ 0.5, glucose 16. The cerebellum was dissected free and 300 μm slices were cut along the folia, perpendicular to their surface, to obtain long uncut parallel fibers. Hippocampal 300 μm slices were cut transverse to hippocampus' longitudinal axis. For recording from alveus fibers, hippocampus was split longitudinally approximately along the fissure separating dentate gyrus from the CA1 area. The slices were cut with a VF‐200 (Precisionary Instruments Inc., San Jose, CA) or, in a few cases, with a Leica VT1200 (Leica Microsystems GmbH, Wetzlar, Germany) and were stored in an oxygenated chamber for at least 0.5–1 h at room temperature before recording. The ionic composition of ACSF used for storage and recording was (in mmol/L): NaCl 125, KCl 1.25, KH_2_PO_4_ 1.25, MgCl_2_ 1, NaHCO_3_ 25, CaCl_2_ 2 and glucose 16 (pH = 7.4). Fast synaptic potentials were always blocked, in all experiments, by adding DNQX (30 μmol/L), APV (50 μmol/L), and picrotoxin (25 μmol/L) to the ACSF.

During recording, the slices were kept submerged in a 3 mL tissue chamber with oxygenated ACSF flowing at 4–7.5 mL/min. The temperature was controlled by a temperature controller (MTC‐20/2SD, npi electronic GmbH, Tamn, Germany).

### Drugs

6,7‐dinitroquinoxaline‐2,3‐dione (DNQX) and (DL)‐amino‐5‐phosphonovaleric acid (APV), were purchased from Tocris Bioscience, (Bristol, UK). Picrotoxin, tetraethylammonium (TEA), and salts were purchased from Sigma–Aldrich (Milwaukee, WI).

### Compound action potentials recordings

**Figure 1:**
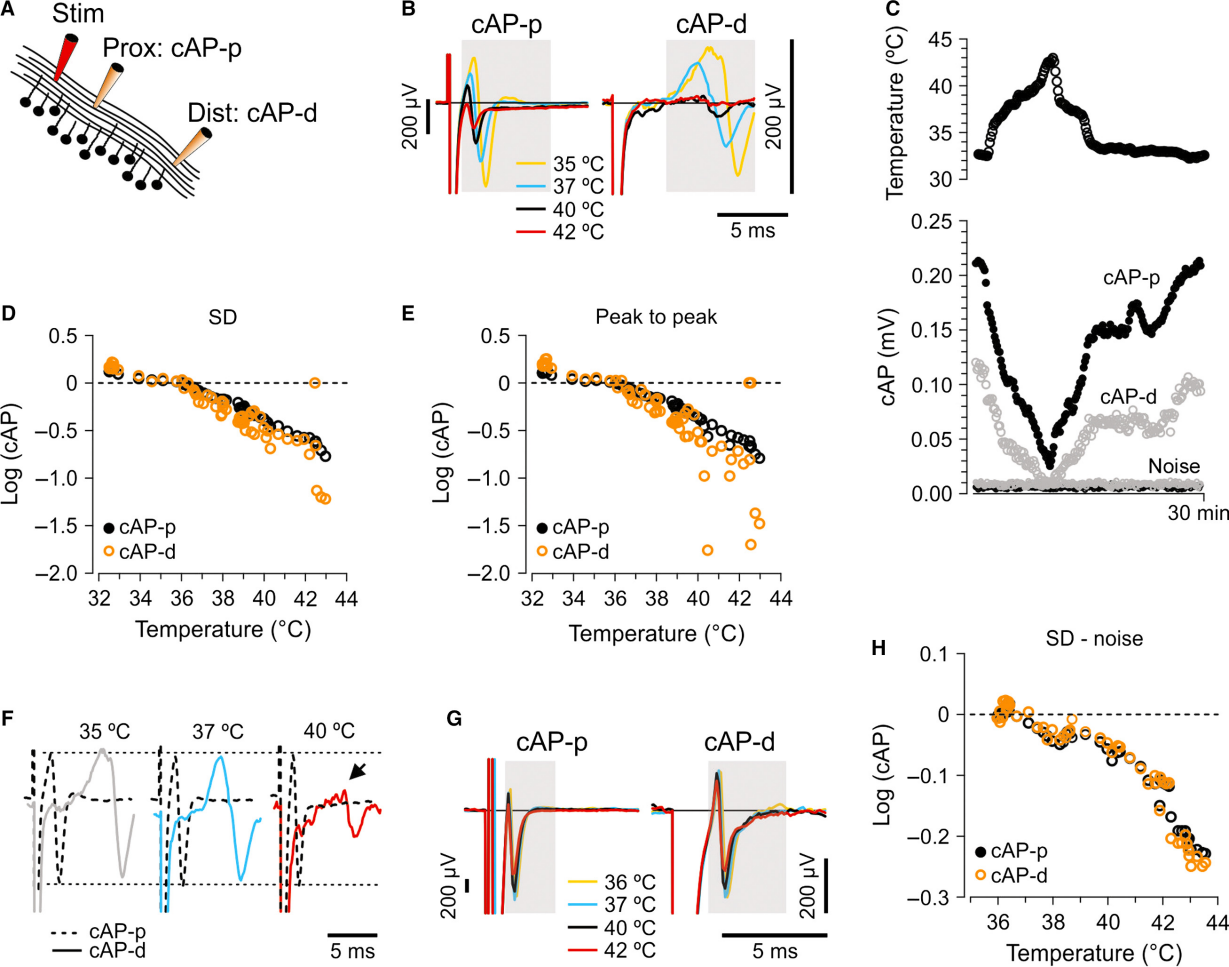
Detection of conduction failures in parallel fibers, single‐experiment examples. (A) Two recording electrodes were positioned along the parallel fibers to record cAP at different distances (cAP‐p and cAP‐d: proximal and distal compound action potential, respectively) from the initiation point (stimulating electrode, Stim). The distance between Stim and proximal electrode (Prox) was always shorter than between Prox and distal electrode (Dist). (B) cAP‐d was usually smaller than cAP‐p at all temperatures (note different scaling). Additionally, cAP‐d decreased more than cAP‐p at high temperatures. Signals have been scaled to the same peak‐to‐peak amplitude at 35°C to better compare amplitude drops at high temperatures. Shaded area indicates a time window bracketing the cAP (appearing at all temperatures during the experiment) in which the magnitude of the volley was measured. The noise was measured at the beginning of the trace, within the same time window as window bracketing the cAP for each individual electrode. (C) Temperature increase (top panel) from 33 to 44°C resulted in concomitant reduction of amplitudes at both proximal and distal recording sites (lower panel). The cAPs increased again as temperature was reduced to 33°C. The noise measurement is shown separately and was in this case not subtracted from the cAP values. (D) cAP magnitude measured as SD of the signal in the time windows indicated in (B). cAP‐d (orange) dropped more than cAP‐p (black) at temperatures ˃37°C, plotted as logarithmic values, and normalized to their average values at 36°C. (E) Amplitude of cAP measured as peak‐to‐peak of cAP, in the same experiment as in B–D, showed similar results as when signal was measured as SD. (F) Example traces from 35, 37, and 40°C (same as in panel B), normalized to the cAP‐p peak‐to‐peak values (so that cAP‐p, dashed line, has the peak‐to‐peak value of 1.0), show a drop of cAP‐d amplitude relative to cAP‐p at 40°C (arrow). (G) Example of signal recorded from myelinated fibers of alveus. As in panel B, shaded area indicates a time window bracketing the cAP (appearing at all temperatures during the experiment) in which the magnitude of the volley was measured. In contrast to unmyelinated axons in cerebellum, cAP‐p and cAP‐d from the myelinated axons showed proportional drop during increase in temperature. (H) cAP magnitude of the signal shown in G confirmed the lack of selective drop in cAP‐d (orange) when compared with cAP‐p (black) while increasing temperature. Measurements were normalized to their average at 36°C, and plotted as logarithmic values.

Compound action potentials (cAPs) were recorded in cerebellum from parallel fibers, and in hippocampus from stratum radiatum and alveus fibers. The recording electrodes were pulled to a tip size of ~30–60 μm in diameter, from borosilicate glass capillaries (BioMedical Instruments, Germany). Outer and inner diameters were 1.5 and 0.86 mm, respectively. A proximal recording electrode was positioned, 0.3–0.8 mm from the stimulating electrode, and the distance between the proximal and distal recording electrodes was 0.5–2.1 mm (Fig. 1A).

Axons were activated by short electrical pulses (50–150 μsec) from an ISO‐stim 01MD (npi electronic GmbH, Tamm, Germany), delivered through a monopolar glass pipette with a tip diameter of 30–70 μm. Trains of stimuli with identical intervals of 10, 20, or 30 msec were repeated every 10–20 sec, although some experiments used single stimuli repeated every 10–20 sec. When amplitude and latency were stable at a temperature around 34–36°C, evaluated >5 min, temperature was increased, approximately 0.5–1.6°C per minute until it reached 43–44°C, or alternatively until cAP amplitudes were undetectable. The temperature was then reduced to 34–36°C.

### Grease‐gap recordings

To monitor changes in the axonal action potential with its depolarizing after‐potential (DAP) during fever‐like temperatures, we used the grease‐gap technique as previously described (Palani et al. 2012; Pekala et al. 2014). Briefly, a grease‐gap pipette was made from pipette tips, usually 20 μL (Molecular BioProducts, 3521) and the tip cut to make an opening of ~1.5 mm. An arch, ~0.5 mm wide and 0.3 mm high, was carved out at the bottom. The cut surface of the pipette along with the arch was covered with grease (petroleum jelly, Vaseline, Chesebrough‐Pond's Inc.). The bottom of the plastic recording chamber was covered with Sylgard 184 (Dow Corning, Auburn, MI). By positioning the arched window over a bundle of axons, we could record a monophasic signal with distinct fast and slow, positive components, corresponding to a compound Na^+^‐dependent spike and its DAP, respectively.

For ordinary extracellular and grease‐gap recordings, we used a DPA‐2FL amplifier (npi electronic GmbH, Tamm, Germany) with a 10× at the head‐stage gain. Signals were amplified 100× at the amplifier (with the head stage gain that is 1000×), low pass filtered at 1 kHz, digitized at 10 kHz with National Instruments NI USB 6343, and stored on computer hard disk.

### Whole‐cell patch‐clamp recordings

To record from cerebellar granule cells, we used the blind approach (Blanton et al. 1989) as described previously (Palani et al. 2012; Pekala et al. 2014). Pipettes were pulled from borosilicate glass (OD 1.5 mm ID 0.86 mm, BioMedical Instruments, Germany) and had tip resistance of 5–8 MΩ. The intracellular solution contained (in mmol/L): K‐gluconate 120, KCl 20, MgCl_2_ 2, Na_2_ATP 2, HEPES 10, pH 7.2. Intracellular patch‐clamp recordings were amplified by SEC‐10LX (npi electronic GmbH, Tamm, Germany), voltage clamped in linear mode, current clamped in switching mode. The electrode's access resistance, between 6 and 20 MΩ, was not compensated. Axons were electrically activated by a 20–50 μm wide monopolar glass electrode.

### Analysis

#### cAP magnitude

To measure the cAP, we used the standard deviation (SD) of the trace in a time window bracketing the action potential volley appearing at all temperatures during the experiment (gray area in Fig. 1B and G). The window is necessary because the latency shifts with temperature. Using the maximal peak‐to‐peak amplitude within a time window has the disadvantage of picking up the maximal peak‐to‐peak noise when cAP amplitudes are small. In experiments with relatively large cAP amplitudes, the peak‐to‐peak measure gave results similar to SD (Fig. 1D and E). However, because the amplitude often dropped considerably, we used SD as the magnitude of the cAP.

For the analysis of temperature effects on the cAP amplitudes during the four‐stimuli trains, we used measurement from the distal electrode because they were always well separated from the stimulus artifact, and would theoretically, be most influenced by conduction failures due to their longer conduction distance.

#### Noise subtraction and cutoff

Baseline noise was measured at the beginning of the sweep, separately at proximal and distal electrode, in a time window similar to the one used to measure the cAP. This noise (SD_noise_) was subtracted from cAP (SD_cAP_), according to the equation: sqrt(SD_cAP_^2^ − SD_noise_^2^). The term “cAP magnitude” is used throughout this article for the magnitude of cAP, measured as SD, with subtracted noise. We included only recordings with detectable signals at *both* proximal and distal electrode, leaving data with a high signal‐to‐noise ratio of the noise‐subtracted cAP at both electrodes, signal‐to‐noise ratio = 26.5 ± 2.8 and 3.4 ± 0.4 on average at the proximal and distal electrodes, respectively, in the 40–42°C range. The strategy to exclude recordings with undetectable responses resulted in exclusion of many recordings that had undetectable responses only at the distal electrode at high temperature, and many of these probably were due to conduction failures. Therefore, this strategy most likely reduced, but could not have increased, the opportunity to detect conduction failures.

#### cAP latency

Latency was measured for each response in the train of four stimuli as the time from the beginning of the stimulus artifact to the time of the minimum of the biphasic cAP.

#### Temperature binning

All noise‐subtracted cAPs and their latencies were ordered with respect to the temperature at which they were taken, collected in 1°C bins, and the median value of each temperature bin was used for further calculations. The temperature of a temperature bin is given as its lower limit + 0.5°C, for example, the bin of 37–38°C is called “37.5°C”.

#### Grease‐gap recorded cAP and DAP

The amplitude of the cAP was measured as a maximum value of cAP relative to the baseline. The DAP was measured 3 msec before the artifact of the following stimulus, to obtain DAP amplitude immediately before the next cAP.

### Statistics

**Figure 2:**
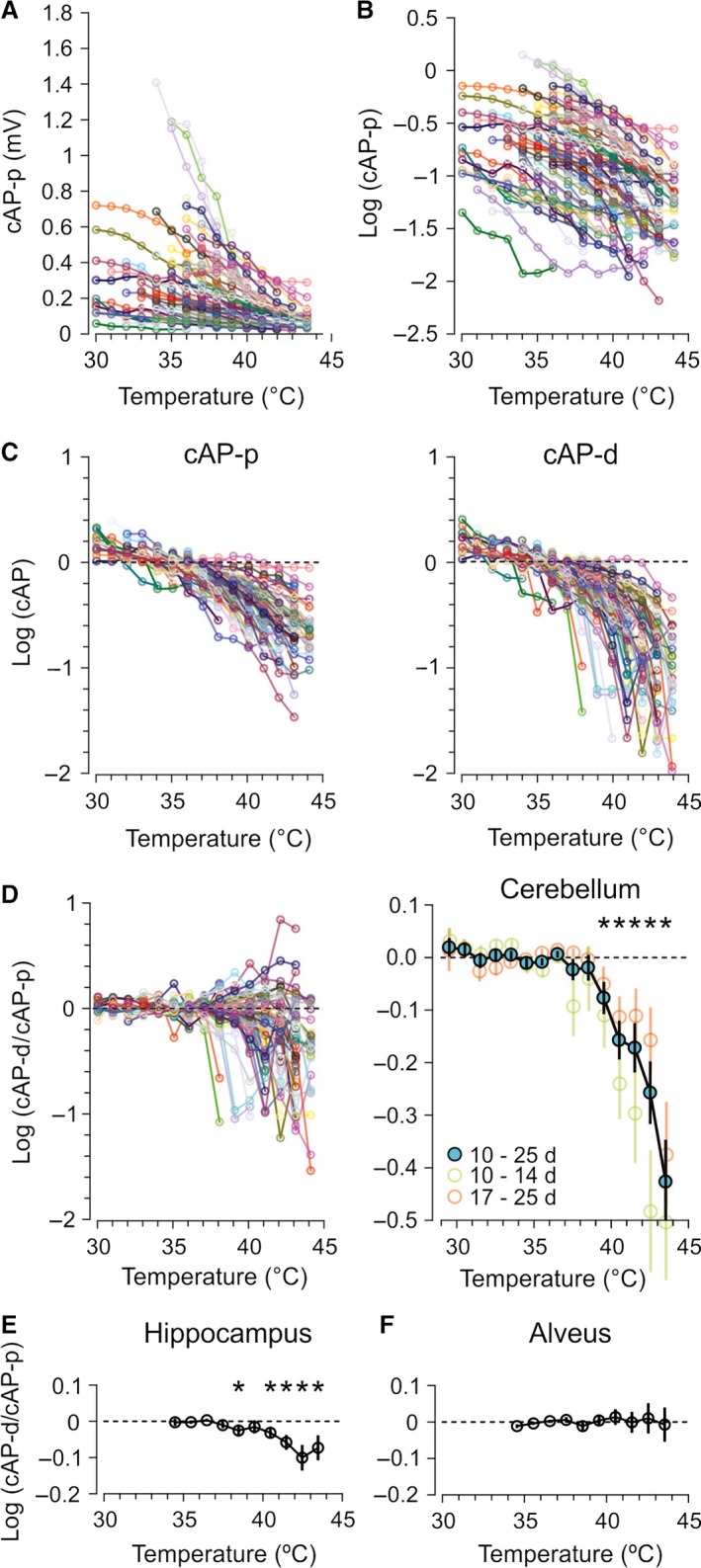
Effect of temperature on cAP‐p and cAP‐d in cerebellum (A–D) and hippocampus (E–F). (A) The effect of temperature on cAP‐p in 78 recordings marked by different colors. (B) The logarithmic (base 10) values of the same data as in (A). (C) The left plot shows individual experiments (the same as in A and B) normalized to the average of the data points from 30 to 37°C. The same procedure was applied to cAP measured at the distal electrode (right). Note the larger drop of cAP‐d compared to cAP‐p at high temperatures. (D) The difference in the drop of cAP‐d and cAP‐p expressed as logarithms of the ratio cAP‐d/cAP‐p, in 78 individual experiments (left). The dominating negative values at high temperatures show that cAP‐d fell more than cAP‐p. The average of all experiments (right) confirms that cAP‐d dropped more than cAP‐p at temperatures ≥39.5°C (*P =* *0.012 at 39.5°C, and *P *<* *0.0003, at temperatures >39.5°C, *n *=* *78). Splitting the data in groups from 10 to 14 and 17–25 days old (none were 15 or 16 days old) showed that the cAPs fell more at distal electrode both in the youngest and oldest groups, with a tendency to drop most in the group of young animals. (E) Similar to cerebellar parallel fibers, hippocampal unmyelinated axons in stratum radiatum of the Ca1 area also showed larger drop of cAP‐d than cAP‐p at high temperatures. However, the magnitude of the drop was smaller than in cerebellum (*P <* *0.03, *n *=* *25). (F) Myelinated axons in alveus did not show changes of the cAP‐d/cAP‐p ratio, suggesting that these axons did not fail in the tested temperature range. (*P *>* *0.05 at all temperatures, *n *=* *16).

**Figure 3:**
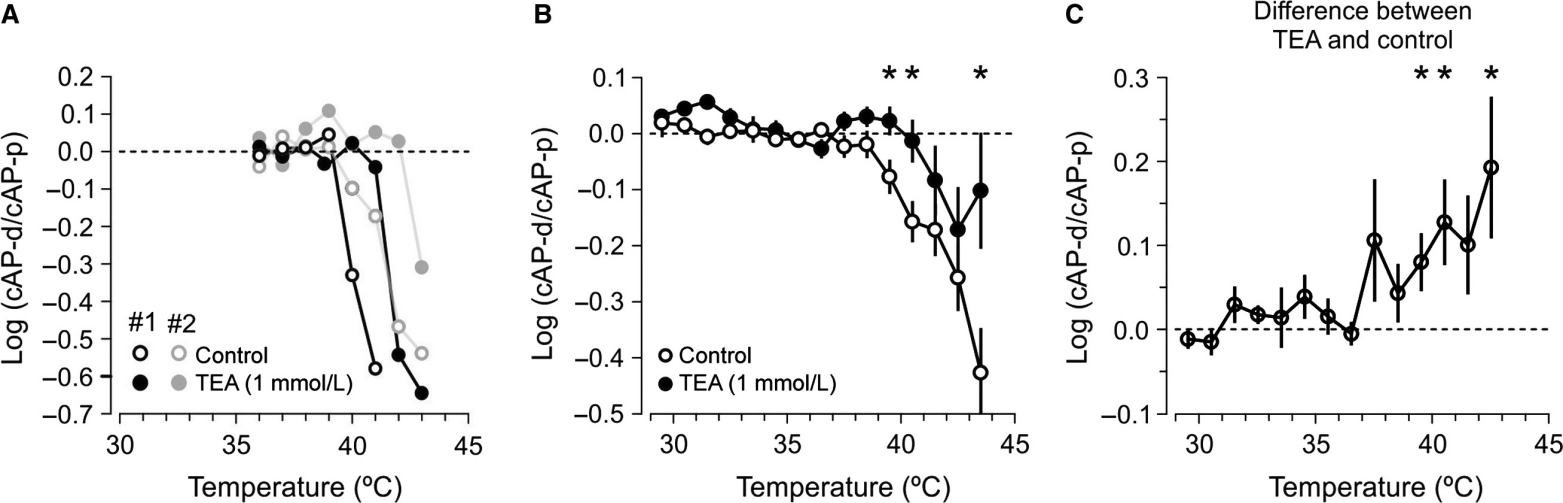
Effect of TEA (1 mmol/L) on spike conduction in parallel fibers. (A) Examples of preferential drop of cAP‐d during elevated temperatures, measured in two individual experiments (black and gray) before (open circles) and during application of TEA (filled circles). Note a shift toward higher temperatures in the drop of log (cAP‐d/cAP‐p) in the presence of TEA. (B) Average values of log (cAP‐d/cAP‐p) from 16 experiments with TEA in the bath (filled circles), compared to 78 experiments without TEA (open circles). (* indicates *P *<* *0.05 for the difference between TEA and control). (C) The failure‐reducing effect of TEA shown by the average of the differences between log‐ratios before (control) and during TEA (*P *=* *0.01, 0.01, 0.05, and 0.02 at 39.5, 40.5, 41.5, and 42.5°C, respectively, *n *=* *15).

Data are presented as mean ± SEM unless otherwise specified. To compare log‐ratios, like in Figure 3B, we used Student's *t*‐test, two‐tailed, assuming unequal variance. Significance of changes in parameters between three or more groups of means (like in Figs. 5, 7) were tested with one‐way ANOVA, followed by Fisher's least significant difference (LSD) multiple comparisons test. To determine if log‐ratios were different from 0, as in Figures 2D, E, and F, 3C, and 4E, we used one‐sample *z*‐test. Statistical analyses were performed in Excel or IBM SPSS Statistics 22. *P* < 0.05 were considered significant.

**Figure 4:**
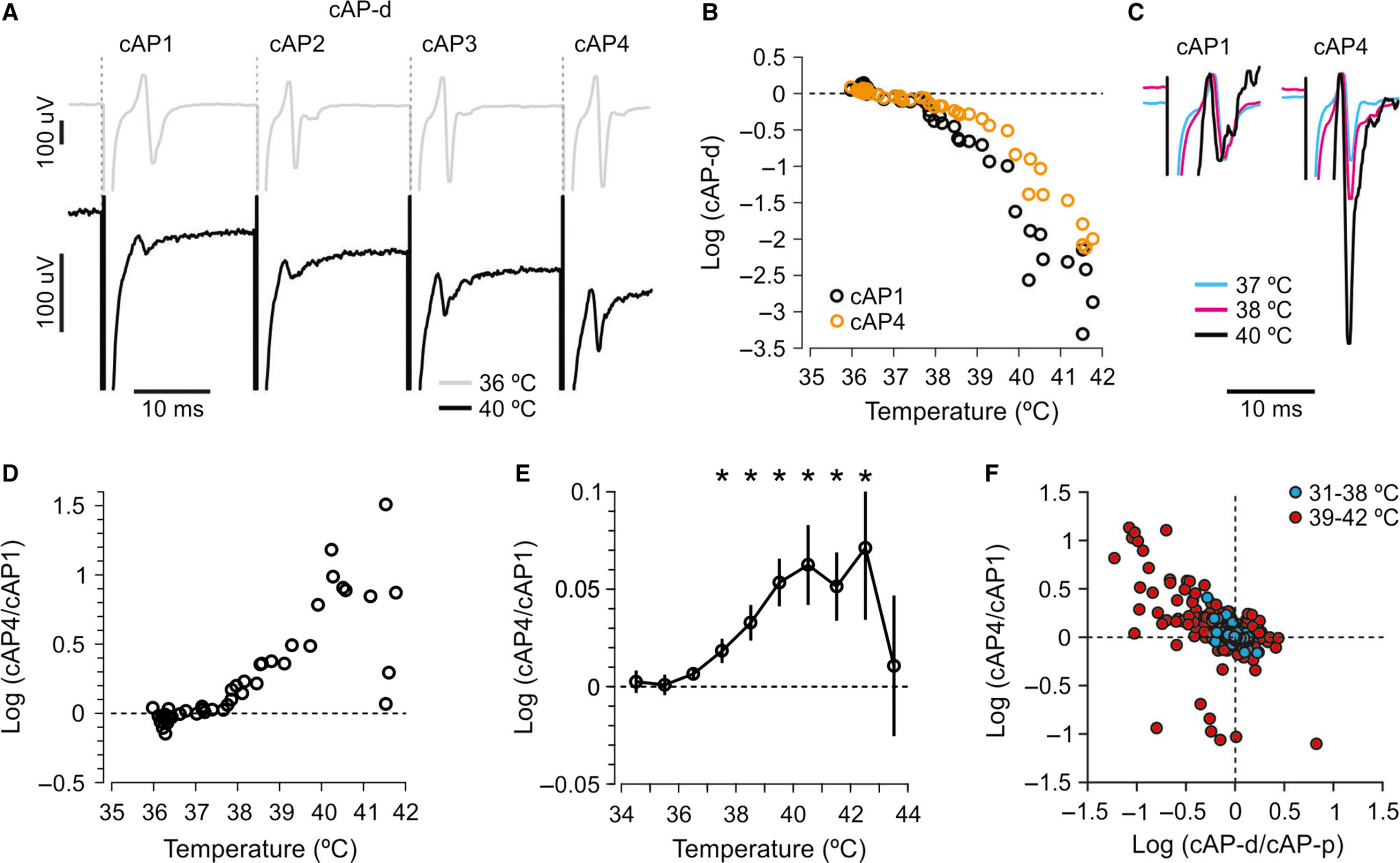
Effects of temperature on cAPs during a train of spikes. (A) Four stimuli were repeated at 20 msec intervals. The amplitude of all cAPs (cAP1–4) in the train decreased during temperature increase from 36°C (gray trace) to 40°C (black trace). Note the difference in calibration bar. (B) Measurements of cAP1 and cAP4 (the same experiment as in A) showed that cAP4 (orange) declined less than cAP1 (black) at temperatures ˃38°C. (C) cAP4 normalized to cAP1 peak‐to‐peak amplitude at 37, 38, and 40°C, to illustrate the reduced drop of cAP4 relative to cAP1 at high temperatures (the same experiment as in A and B). (D) Logarithm of the cAP4/cAP1 ratio shows that cAP4 dropped less than cAP1 at temperatures ˃38°C. (E) The average of 76 experiments similar to the one displayed in panels A–D shows that cAP4 dropped less than cAP1 at temperatures >37.5°C (*P <* *0.02, *n *=* *76). (F) Smaller values of cAP‐d/cAP‐p (interpreted as more failures) were associated with larger values of cAP4/cAP1 (interpreted as fewer failures after at least one spike). This association was much stronger at high than low temperatures (3–6 data points from each of 76 experiments).

## Results

### Conduction failures at moderately elevated temperatures in cerebellum and hippocampus

To detect conduction failures, we recorded compound action potentials (cAPs) from cerebellar parallel fibers with two recording electrodes positioned at different distances along the axonal path (Fig. 1A). Fast synaptic signals were blocked in all experiments presented in this article (see Methods). Elevation of the bath temperature from 33 to 42°C reduced the cAP amplitudes at both electrodes (Fig. 1B). The cAPs increased again as temperature was reduced to 33°C, showing that the amplitude drop was not caused by irreversible damage (Fig. 1C).

The observation that the cAP almost disappeared at the highest temperatures is likely due to failure to activate or failure to propagate the spikes. To distinguish those two possibilities, we compared the reduction of the proximal and distal cAP (cAP‐p and cAP‐d, respectively). The cAPs were measured as the SD of the response in a time window that bracketed the cAP at all temperatures (gray area in Fig. 1B and G), and noise was subtracted, detailed in Methods. Because cAP‐p was usually larger than cAP‐d, we normalized them to their average values <37°C. We could then see that cAP‐d dropped more than cAP‐p at temperatures >37°C (displayed as logarithmic values from a typical experiment in Fig. 1D). The measurement of cAP as peak‐to‐peak amplitude (Fig. 1E) within the gray window gave similar results as SD at high amplitudes, but because of better performance at low amplitudes, as explained in Methods, we used SD for the remaining experiments. The bigger drop in amplitude of cAP‐d, relative to cAP‐p, at high temperatures was also illustrated by normalizing both cAPs to cAP‐p (so that cAP‐p has the peak‐to‐peak value 1.0) at 35, 37, and 40°C (Fig. 1F). Myelinated fibers in alveus (Fig. 1G) did not show a selective drop in cAP‐d, when compared with cAP‐p (Fig. 1H). This suggests that the temperature conduction failures were specific for thin, unmyelinated axons.

The temperature‐sensitive drop of cAP‐d found in unmyelinated fibers of cerebellum was confirmed by comparing cAP‐p and cAP‐d in 78 experiments from rats 10–25 days old (average 17.9 days) at different temperatures. Increasing temperature reduced all cAPs to very small values (Fig. 2A), displayed in Figure 2B as logarithmic values. Similar to the example in Figure 1D, we normalized cAP‐p and cAP‐d to their average temperatures <37°C (Fig. 2C). Visual inspection of cAP‐p and cAP‐d from all experiments suggested that increasing temperature reduced cAP‐d more than cAP‐p (Fig. 2C).

To evaluate if cAP‐d dropped more than cAP‐p, we calculated the ratio cAP‐d/cAP‐p in a range of temperatures (Fig. 2D). The average of these ratios showed that cAP‐d dropped more than cAP‐p at temperatures >39°C (first temperature bin significantly < 0, *P *=* *0.013, was the bin 39–40°C). This preferential distal‐recording (cAP‐d) drop contrasted with the proportional drop of cAP‐p and cAP‐d at temperatures <39°C (values around zero in the logarithmic plot in Fig. 2D) and strongly suggested that some spikes failed to propagate between the two recording electrodes at temperatures >39°C.

The effect was not isolated to the youngest animals because the groups 10–14 and 17–25 days old, both showed significant drops in cAP‐d/cAP‐p ratio at high temperatures (Fig. 2D, right), although the youngest group was slightly more temperature sensitive because their cAP‐d/cAP‐p ratio dropped significantly >39°C while the oldest group dropped significantly >40°C.

The temperature‐sensitive conduction failures may be typical for gray matter axons because such axons in hippocampus also showed preferential drop at the distal recording electrode, at temperatures >38°C (Fig. 2E, average age 20.5 days, *n *=* *25), although of smaller magnitude than in the parallel fibers in cerebellum. Furthermore, thin, unmyelinated CNS axons may be uniquely temperature sensitive because myelinated CNS axons (hippocampal alveus fibers) did not show signs of failures at comparable temperatures (Fig. 2F, average age 21.4 days, *n *=* *16).

### Block of voltage‐sensitive K^+^ channels reduced temperature‐induced failures

One of the hallmarks of conduction failures is that spike broadening, either by reducing temperature or by drugs, help overcome some of the failures (Bostock et al. 1978; Westerfield et al. 1978; Swadlow et al. 1980). We tested this idea using 1 mmol/L TEA in the extracellular solution, shown to efficiently block fast Kv3 channels (Rudy and McBain 2001) and also known to broaden the spike in cerebellar parallel fibers (Sabatini and Regehr 1997; Matsukawa et al. 2003; Pekala et al. 2014). Figure 3A presents two typical experiments with approximately 2°C higher threshold for conduction failures when exposed to TEA.

On average, there were fewer conduction failures at 39.5 and 40.5°C with TEA than without (Fig. 3B). The average may underestimate the TEA effect because the drop in cAP‐d/cAP‐p ratio occurred at slightly different temperatures in different experiments (like the two illustrated in Fig. 3A). We therefore calculated the difference between log (cAP‐d/cAP‐p) with and without TEA in the individual experiments. The average of these pairwise differences confirmed that TEA significantly increased the threshold for conduction failures at 39.5 and 40.5°C (Fig. 3C). At higher temperature (41.5°C), the variability prevented clear significance (*P *=* *0.053), which may be due to the fact that cAP magnitude was very small, and also that conditions for spike conduction deteriorated beyond rescue.

The finding that 1 mmol/L TEA reduced the preferential distal‐electrode drop supports the hypothesis that voltage‐sensitive potassium channels (Kv) contributed to the conduction failures observed at high temperatures, although TEA may have reduced failures caused by other mechanisms too.

### Do spikes improve the conduction fidelity of the following spikes?

Many thin, unmyelinated CNS axons have a depolarizing after‐potential (DAP) that offers an explanation of the hyper‐excitable period following individual spikes in such axons (Gardner‐Medwin 1972; Wigstrom and Gustafsson 1981; Palani et al. 2012). We therefore tested if the DAP could help spikes propagate at high temperatures.

We repeated the parallel fiber stimulus four times with 20 msec intervals, which is within the duration of the DAP (Palani et al. 2012; Pekala et al. 2014) and within the range of granule cell firing frequencies in vivo (Eccles et al. 1966; Chadderton et al. 2004; Jorntell and Ekerot 2006). With increasing temperature, all four cAPs declined. The first response at the proximal and distal electrodes were analyzed previously (Figs. 1 and 2) and showed that cAP‐d fell more than cAP‐p at temperatures >38.5°C, interpreted as conduction failures. Interestingly, during the train of stimuli, we observed that the last cAP (cAP4) in the train declined less than the first cAP at elevated temperatures (cAP1, shown at 36 and 40°C in Fig. 4A from a typical experiment).

The reduced drop of cAP4 relative to cAP1 was quantified similarly to the analysis of cAP‐p and cAP‐d (details in Methods). First, we normalized cAP1 and cAP4 to their average values <37°C, showing that cAP1 fell more than cAP4 at temperatures >38°C (Fig. 4B, same experiment as in A).

Example traces from 37, 38, and 40°C, normalized to the cAP1 peak‐to‐peak values (Fig. 4C) show that relative to cAP1, cAP4 was much larger at 38 and 40°C, meaning that cAP4 fell less than cAP1 at those temperatures. Similarly, the cAP4/cAP1 ratio showed that cAP4 declined less than cAP1 at temperatures >37°C (Fig. 4D). On average, the cAP4/cAP1 ratios increased significantly at temperatures from 37.5 to 41.5°C (Fig. 4E). A possible explanation for this is that there were fewer conduction failures at cAP4 compared to cAP1 at the high temperatures because this was in the temperature range in which conduction failures were detected by the cAP‐d/cAP‐p ratio (Fig. 2). If so, larger values of cAP4/cAP1 would be expected to be associated with the smaller values of cAP‐d/cAP‐p, and more so with increasing temperature, exactly as seen in Figure 4F (*n *=* *78).

Theoretically, excitability may have increased during the four‐stimulus train due to accumulation of extracellular K^+^. If more K^+^ accumulated at high than low temperature, it may offer an explanation for the temperature‐sensitive correlation shown in Figure 4F. However, based on the results described in the following section, we find that unlikely.

### Latency changes suggested an excitability increase of constant time course and magnitude following each spike

**Figure 5:**
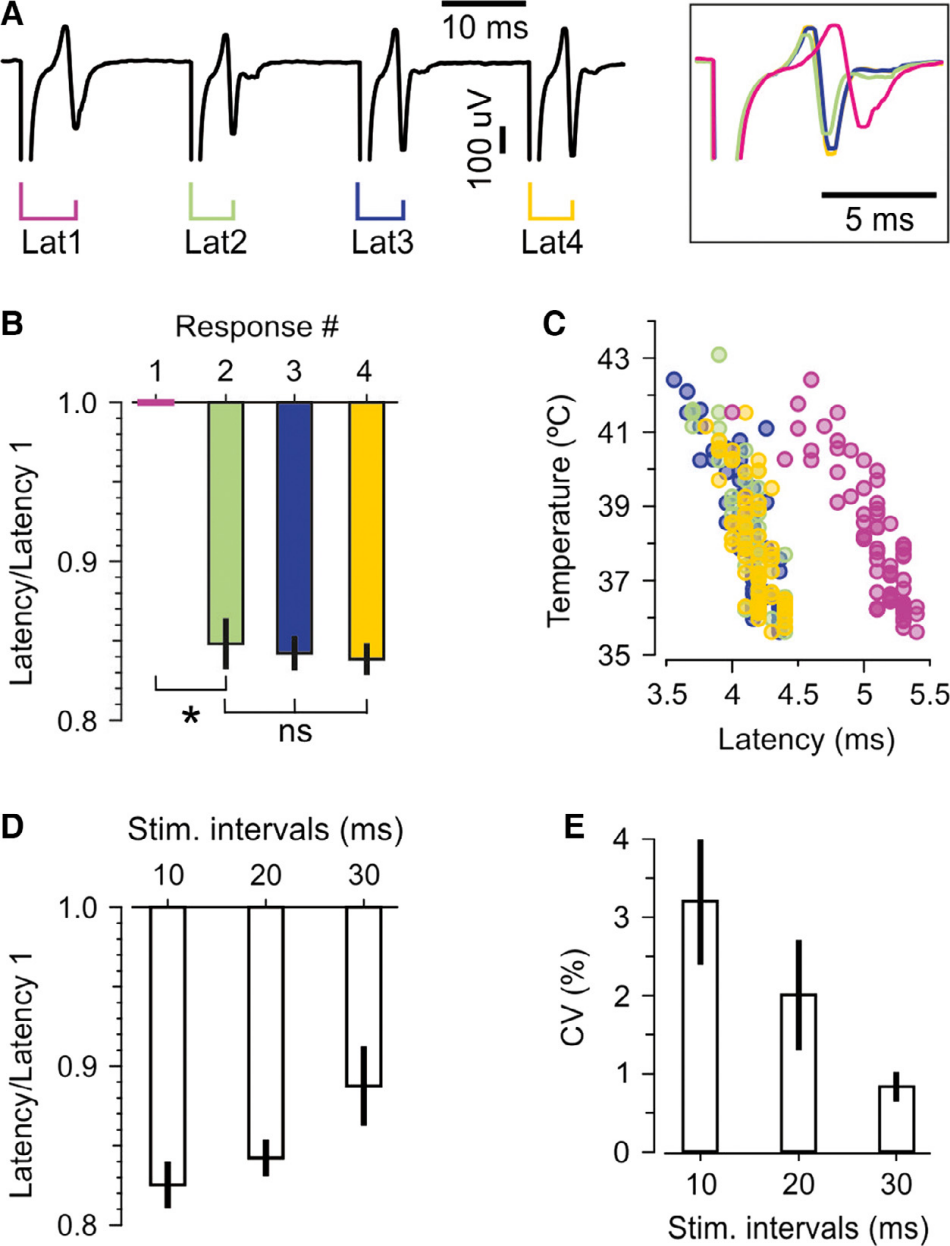
Latency of cAPs in response to repeated stimuli, recorded at distal electrode. (A) Four cAPs in response to four equal stimuli given with 20 msec intervals, at 37°C. Compared to the first latency (Lat1), the second latency (Lat2) was shorter, but the second, third (Lat3), and fourth (Lat4) latencies were very similar. The similarity of Lat2‐4 is presented also by aligning the four cAPs to their stimulus artifacts (inset on the right). Color codes as in latency markers in left hand‐side panel. (B) Lat2‐4 normalized to Lat1, in 20 experiments at 37°C, with 20 msec stimulus intervals, shows that the latency was shorter after the first cAP, but changed little at the three last cAPs in the four‐stimulus trains. *P <* *0.0001, ns: *P *>* *0.6. *n *=* *20. (C) The latencies of all four responses (the same recording as in A) decreased as temperature increased. (D) Average relative latencies at 37°C with 10, 20, and 30 msec stimulus intervals shows reduced effect on latency at longer intervals. The average of Lat2‐4 from each experiment was used (*n *=* *22, 20, and 13 at intervals 10, 20, and 30 msec, respectively, *P *=* *0.04). (E) The CV of Lat2–4 for the same intervals and experiments as in D. (*n *=* *22, 20, and 13 at intervals 10, 20, and 30 msec, respectively, *P *<* *0.05).

A spike with a DAP would be expected to speed up conduction of the spike that follows at its tail. This was exactly what we observed, the latencies of the second (Lat2), third (Lat3), and fourth (Lat4) cAPs were shorter than the latency of first (Lat1) cAP (interval = 20 msec, at 37°C, Fig. 5A and B). The reduced latency already at Lat2 confirmed that the axons were more excitable after being activated once (Gardner‐Medwin 1972; Wigstrom and Gustafsson 1981; Soleng et al. 2004). This effect did not accumulate during the train, observed as overlap of the three last responses (Fig. 5A, right) and was confirmed by similar reduction of Lat2‐4 in 20 experiments (at 37°C, Lat2‐4 were: 85 ± 2%, 84 ± 1%, and 84 ± 1% of Lat1, respectively, Fig. 5B).

Although the latency to each cAP was shorter at higher temperature, the relative reductions in Lat2‐4 were similar (Fig. 5C). At 40°C, Lat2‐4 were 89 ± 3%, 91 ± 3%, and 89 ± 3% of Lat1, respectively (all *P *<* *0.003). Lat2‐4 were not significantly different from each other (all *P *>* *0.4, *n *=* *20, data not shown).

Such a very stable reduction of Lat2‐4 is unlikely to be due to factors that accumulate with repeated spikes, such as extracellular K^+^, unless the maximal velocity was already reached at Lat2 (a “ceiling effect”). To test for a ceiling effect, we used different stimulus intervals (10, 20, and 30 msec). We found that the maximal velocity was not reached because the different intervals resulted in different latency changes. Average values of Lat2‐4 were different, 82 ± 1%, 84 ± 1%, and 89 ± 2% of Lat1, for 10, 20, and 30 msec stimulus intervals, respectively (Fig. 5D).

At the longest intervals (30 msec), the latency reduction was not only the smallest but also had the lowest variability between Lat2‐4 (Fig. 5E, coefficient of variation 3.2 ± 0.8%, 2.0 ± 0.7%, 0.8 ± 0.2% at 10, 20, and 30 msec intervals, respectively). This is opposite of what would be expected by a ceiling effect, which would occur at the shortest latency when the axon could not conduct faster. We therefore conclude that the reduced latency was due to an excitability‐increasing process of constant time course and magnitude, relatively activity independent.

### The DAP in parallel fibers helps propagate spikes

**Figure 6:**
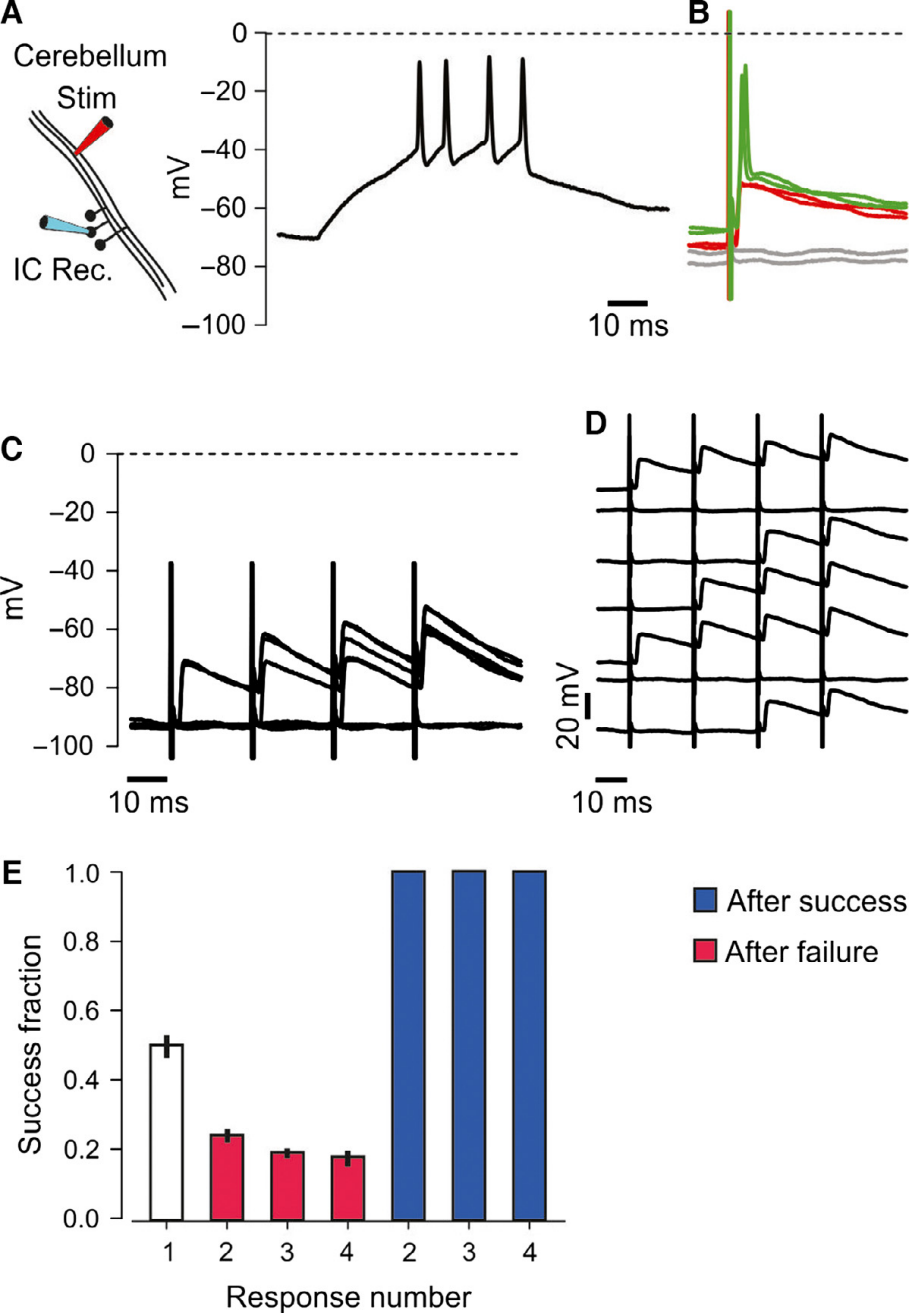
Antidromic failures were reduced by the axonal DAP. (A) Intracellular tight‐seal recording from cerebellar granule cell in a 24‐day‐old rat. This and all cells accepted for analysis responded with spikes to positive current injection, in this case, a 50 ms long 10 pA square pulse. (B) In response to electrical activation of the recorded cell's parallel fiber, antidromic spikes (green) invaded the soma. The invasion of full antidromic spikes (green) was prevented by hyperpolarization of the soma, and then only attenuated spikes arrived at the soma (red). By further hyperpolarization, also the slower, attenuated spikes failed to invade the soma (gray). Vertical axis is the same as in A. (C) The somatic membrane potential was adjusted to give ~50% invasion failures at the first response in the train of four stimuli. In the presented example, seven consecutive stimuli of equal strength gave five failures and two successes at the first stimulus. (D) The same traces as in C, but vertically separated so that the individual four‐stimulus train can be seen. When spike was detected (full or attenuated, in this example, attenuated), the following stimuli always elicited a spike that propagated far enough to be detected at the soma. (E) The average success fraction at the first stimulus in 16 experiments (open bar). When there was a failure at first, second, or third stimulus, the success rate for the following stimulus did not increase (red bars). However, when a successful spike was detected at the soma at first, second, or third stimulus (blue bars), a success followed almost always (only two failures after totally 1784 successes).

Our hypothesis was that the axonal DAP increased excitability after individual spikes and helped conduction in the temperature range where some axons failed. To test this, we electrically activated the parallel fibers while recording antidromic spikes from the granule cell soma. The recorded cells were assumed to be granule cells based on their location in stratum granulosum, activation from stratum moleculare with a latency, and their high resistance to somatic current injections (Fig. 6A, mean = 2.10 GΩ, SD = 0.72 GΩ, *n *=* *14, similar to e.g., Diwakar et al. 2009). The average latency between stimulus and the peak of the spike was 2.00 msec (SD = 1.39 msec, *n *=* *14, Fig. 6B, green traces).

At moderately hyperpolarized potentials (−72 mV, Fig. 6B*,* red traces) compared to rest (−66 mV, Fig. 6B*,* green traces), the fast component of the spike disappeared, probably because spike conduction failed at some distance from the soma and the fast component was attenuated by the axonal cable (Sheffield et al. 2011). At even more hyperpolarized potentials, also the slow component disappeared (−75 mV, Fig. 6B, gray traces). We adjusted the somatic potential to give ~50% failures. The responses to identical stimuli showed spike‐like all‐or‐none behavior (red and gray traces).

With ~50% failures at a constant stimulus strength, we repeated the stimulus four times and observed that if the axon conducted one spike, the following stimulus always resulted in a successfully conducted spike (Fig. 6C and D). By counting failures and spikes (including attenuated spikes, like the red traces in B), we observed 1216 failures immediately after a failure, but only two failures immediately after 1784 successfully propagated spikes (16 experiments). This confirms that a successful spike (full or attenuated) increased the chance of successful conduction for the following spikes.

Unless a successful conduction occurred, the success rate did not increase during the train (red bars Fig. 6E). This means that extracellular K^+^ or other excitability‐increasing effects from adjacent axons or other cells had minor effects on the excitability compared to the strong effect of a successfully propagated spike which abolished almost all failures at the following stimulus.

A likely explanation for the failure‐reducing effect of a single spike is that residual depolarization (the DAP) left from the preceding spike helped overcome regions of low excitability. The DAP may therefore offer an explanation for the larger cAP amplitudes at the fourth stimulus compared to the first in the temperature range that induced failures (Fig. 4).

### Constant amplitude and waveform of the DAP

**Figure 7:**
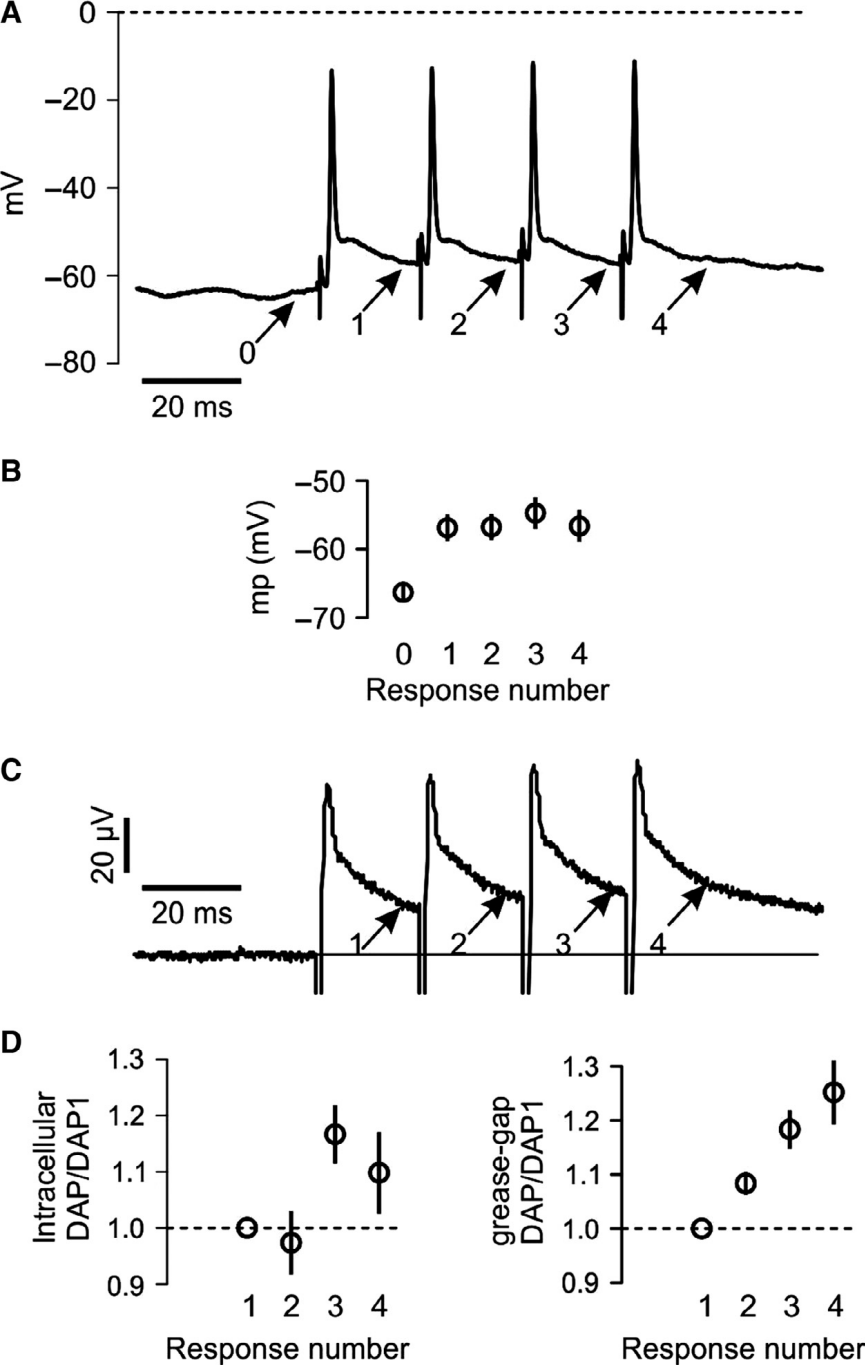
The depolarizing after‐potential is relatively unaffected by moderate spike activity. (A) Example of antidromic spikes recorded at granule cell soma in response to electrical activation of parallel fibers with four stimuli (interval = 20 msec). Membrane potentials were measured before (marked 0) and after stimuli at intervals corresponding to stimulus intervals (marked 1–4). (B) Average values of membrane potentials (mp) measured at points indicated by the arrows in A in 14 experiments. Comparing responses 1–4: *P *=* *0.94, *n *=* *14. (C) Grease‐gap recordings from bundles of cerebellar parallel fibers in response to four stimuli with 20 msec intervals. The grease‐gap signals were small, but showed many features similar to the somatic recordings, for example, the slowly decaying DAP after the fast component. (D) To compare the DAP variability in both recording types on a similar scale, we normalized the DAP amplitudes, measured from baseline before the first stimulus, to the value of the first DAP amplitude both in intracellular (*n *=* *14) and grease‐gap recordings (*n *=* *23), left and right graphs, respectively.

For the DAP to be an acceptable explanation for the very similar latency reduction at all three cAPs (Lat2‐4, Fig. 5), the DAP would need to have a relatively constant amplitude at a given interval after each spike, which we will address in this section. The intracellular DAPs of the antidromic spikes, measured on the decay of responses 1–4 immediately before the following stimulus (arrows in Fig. 7A) were very similar during the train of four stimuli (Fig. 7B, −57.0 ± 2.6, −56.7 ± 1.9, −54.7 ± 2.5, and 56.6 ± 2.7 mV). The SD of the membrane potential of those four DAPs in the individual experiments was only 0.75 ± 0.11 mV on average for 14 experiments. That low variability in the membrane potential may explain the similar latency reductions at cAP2–4 (Fig. 5).

Because the initial segment of the axon has other membrane currents, axial resistance, and input resistance than more distal parts (Kole and Stuart 2012) which theoretically could influence our somatically recorded DAPs, we also recorded the waveform of the axonal spikes far away from the soma using a miniaturized grease‐gap method (Palani et al. 2012).

Grease‐gap recordings give information about the temporal changes of the transmembrane voltage, but not the true voltage. Therefore, to compare intracellular and grease‐gap recordings, we used a measure we could obtain from both recording techniques, namely the difference between DAP amplitude and baseline measured before the first stimulus (arrows in Fig. 7A and C). To compare the DAP variability recorded by different techniques on a similar scale, we normalized the amplitudes to the value of the first DAP amplitude, both in intracellular and grease‐gap recordings (Fig. 7D).

The DAP variability between responses 1–4, measured as coefficient of variation (SD/mean) was 0.095 ± 0.019 (*n *=* *14) and 0.090 ± 0.011 (*n *=* *23, *P *=* *0.84, data not shown) for the intracellular and grease‐gap measurements, respectively. This similarity between the variation of the DAPs measured intrasomatically and by the grease‐gap technique supports the hypothesis that both those techniques detect features of the axonal DAP. The fact that the variability was similar also suggests that the axons may have a DAP variability of approximately 0.75 mV, as recorded at the soma, also further away from the soma. Additionally, these data show that the axonal DAP was relatively resistant to moderate activity (the repetition of four spikes).

### Influence of temperature on action potential and membrane potential

When Hodgkin and Katz (1949) described the temperature effects on the propagating spike in the squid giant axon, they noted that increasing temperature caused a small depolarization of the resting membrane potential, but a large amplitude‐ and width‐reducing effect on the spike. This led them to conclude that the spike failure was not simply due to loss of membrane potential, but depended on spike‐specific mechanisms.

Since it has not yet been possible to measure membrane potential directly in thin unmyelinated cortical axons, we measured steady‐state membrane potential at the soma using tight‐seal recordings. However, fast voltage changes in the axon, like the spike, are probably not well represented at the soma. For example, Kv10.1 is located almost exclusively to the axon, and most likely influences spike repolarization, but no effects of this could be detected by somatic recordings (Mortensen et al. 2015). We therefore measured cAP amplitude from the parallel fibers using a grease‐gap method (Palani et al. 2012). Somatic membrane potential and grease‐gap recorded cAP will be influenced by axonal membrane potential and spike amplitude, respectively, although further qualifications will be given in the Discussion.

**Figure 8:**
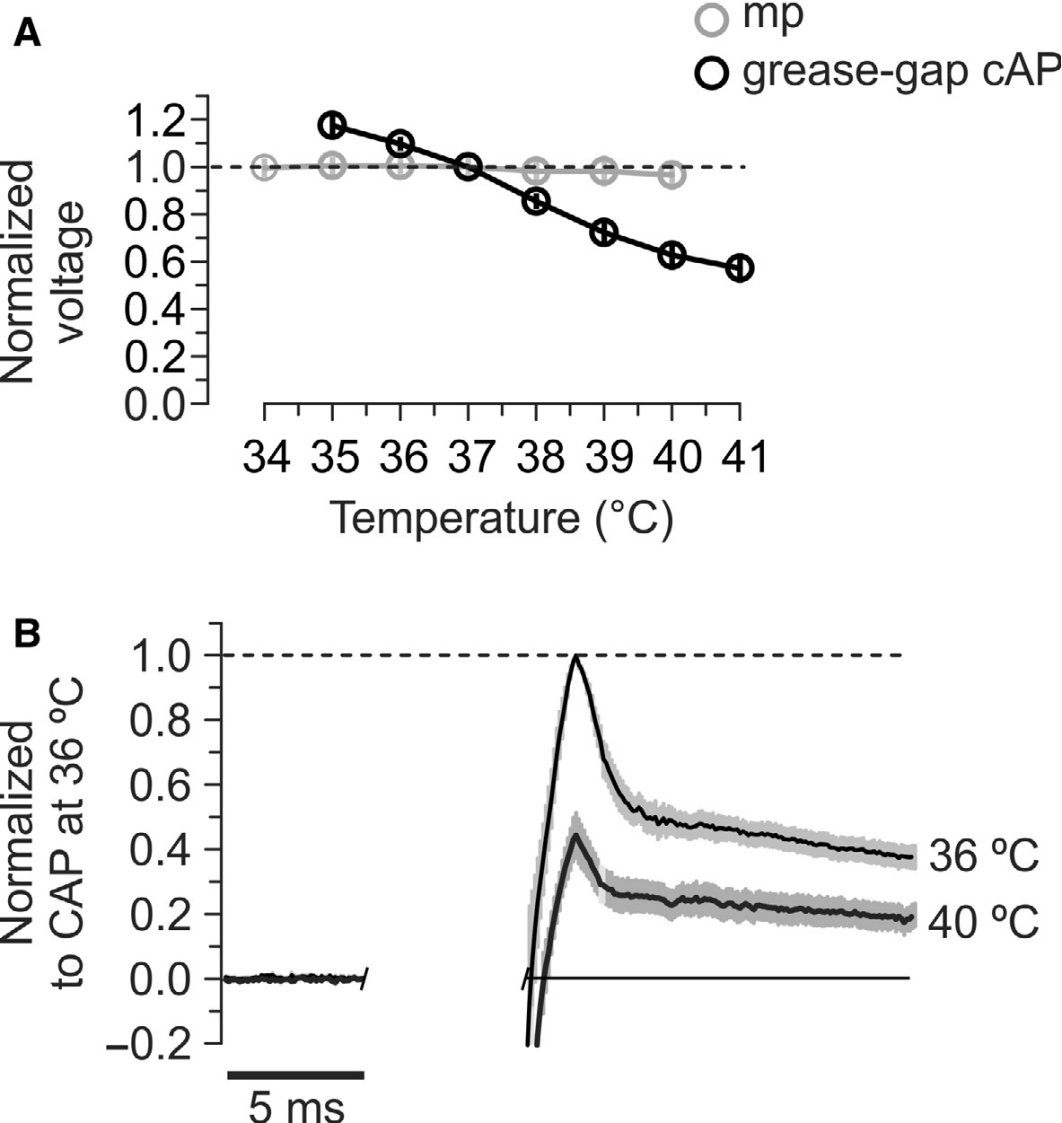
Temperature effects on spike and membrane potential. (A) The membrane potential showed small changes while the grease‐gap recorded cAP clearly lost amplitude as temperature increased. Before averaging, the membrane potentials and peak cAP amplitudes in each experiment were normalized to their values at 37°C. mp – membrane potential, average of eight intracellular somatic recordings from granule cells. Grease‐gap recorded cAP – average of 13 experiments. (B) The average shape (±SEM, shaded area) of the grease‐gap recorded cAP from cerebellar parallel fibers in nine experiments, at 36 and 40°C. Electrical stimulation artifacts were digitally removed. Although the amplitude of the whole signal decreased at high temperature, DAP was still present at 40°C.

The results were qualitatively very similar to Hodgkin and Katz (1949) (Fig. 8A). Membrane potential depolarized by 3% (0.97 ± 0.02, *n *=* *8) at 40°C compared to its value at 37°C (average membrane potential before normalization at 37°C = −66.0 ± 3.4 mV). In contrast, grease‐gap peak cAP decreased by 37% (0.63 ± 0.06, *n *=* *13) at 40°C compared to its value at 37°C.

If the DAP helps to reduce failures of spike propagation during elevated temperatures, as we propose, it should be present also at the temperatures at which axons start to fail. This was indeed the case, as shown by the average shape of potentials from nine grease‐gap recordings at 36 and 40°C, temporally aligned at their peak amplitude (Fig. 8B).

## Discussion

We have identified two intrinsic mechanisms that influence spike propagation in typical gray matter axons: a temperature‐sensitive mechanism that gives conduction failures at temperatures above, but not below normal body temperature, and a temperature and activity‐resistant mechanism that improves conduction fidelity at temperatures immediately above normal body temperature.

### Failures

It is important to distinguish spike conduction and initiation failures because the mechanisms for those failure types are often different. One difference is that conduction failures occur in axons that have an essential function intact, namely the ability to initiate spikes. In general, two recording sites along the axonal path is necessary to identify conduction failures to confirm that axons can initiate spikes, at a proximal site, and to check if the spikes can travel to a more distal recording site.

The two recording sites also gave the opportunity to better interpret the extracellular population recordings, which is still the only practical method to study the electrical behavior of typical mammalian, small‐bouton, unmyelinated cortical axons. For example, increasing temperature reduced the population response (cAP). This may have many causes, like smaller amplitude and width of the individual spikes, and reduced number of axons activated by the electrical stimulus, but such factors would be expected to contribute equally at both proximal and distal recording sites. This assumption was supported by the observation that the cAPs changed proportionally at those two sites at temperatures <38°C for the unmyelinated cerebellar and hippocampal axons. Furthermore, the myelinated alveus axons, measured in exactly the same way as the unmyelinated axons, showed proportional changes at the two electrodes over the full temperature range.

Therefore, when the distal cAP dropped more than the proximal one only in the unmyelinated axons, and only at temperatures >39°C, we concluded that axons failed between the two recording sites. This conclusion assumes that the axons giving rise to the cAPs at the two electrodes had similar properties, but does not require them to be exactly the same axons.

### What caused the failures?

A definite answer requires details about electrical properties *locally* along the axons, which is not available for any axon, but the question has been explored comprehensively with calculations and computer models based on accurate morphological measurements (Westerfield et al. 1978). In general, conduction failures occur because a segment of the axon has low excitability, or because the current from one segment is insufficient to bring the membrane of adjacent segments to threshold. The latter may happen with abrupt changes in diameter, capacitance, or branching, making regions of “impedance mismatch” (Van Essen 1973; Goldstein and Rall 1974; Yau 1976; Luscher et al. 1994).

In combination with such impedance, mismatch increases in temperature and/or activity can cause conduction failures (Rasminsky 1973; Westerfield et al. 1978). We will comment only on the effects of temperature because we deliberately used few stimuli (four) repeated every 20 sec, which is below the activity reported to give activity‐induced failures (Smith 1980; Baginskas et al. 2009).

An important mechanism that contributes to temperature‐induced conduction failures is that increasing temperature reduces the duration and amplitude of the spike (Hodgkin and Katz 1949; Thompson et al. 1985; Yu et al. 2012). These effects are due to faster rate constants of inactivation and activation of the Na^+^ and K^+^ currents, respectively. They become 2.6–3.0 times faster if temperature increases by 10°C (Hodgkin et al. 1952; Schauf 1973; Westerfield et al. 1978). Current amplitude, membrane resistance, excitation threshold, rate of rise of the spike, and cytoplasmic resistivity seem to have less effect (Westerfield et al. 1978; Yu et al. 2012). Such a temperature‐induced reduction in amplitude and/or width is expected to influence the compound action potential, both in the regular extracellular recordings and the grease‐gap recordings, which is compatible with our observation (Fig. 8).

There is, however, a theoretical possibility that temperature causes conduction failures by changing the membrane potential. Depolarization will inactivate Na^+^ channels, and hyperpolarization will increase threshold for spike. From somatic recordings, there are examples of both hyperpolarization and depolarization with increasing temperature, although from low temperatures increasing temperature tend to hyperpolarize (6–33°C in Volgushev et al. 2000), but closer to 36°C, there seems not to be any systematic effect of temperature changes (Thompson et al. 1985; Trevelyan and Jack 2002). Above 36°C, we found a small depolarization (3%, or about 2 mV), similar to Yu et al. (2012), Kim and Connors (2012), and qualitatively similar to Hodgkin and Katz (1949) in the squid axon. Thus, it may be a general tendency to depolarize when temperature deviates from normal body temperature. It is unlikely that the small change in membrane potential we observed would change the spike amplitude enough to cause failures because previously we found that an increase in extracellular K^+^ from 2.5 to 6 mmol/L clearly increased the conduction velocity, meaning that a moderate depolarization gave increased excitability, but the amplitude of the grease‐gap recorded cAP did not change (Pekala et al. 2014).

Our finding that the unbranching myelinated alveus axons did not have conduction failures may, of course, have many causes because electrical properties of myelinated axons are very different from the typical gray matter axons. It is interesting, though, that we have not been able to find any report of conduction failures in normal myelinated, unbranching axons. They do fail to function at high temperatures (Bernstein 1902), but without pathology‐like demyelination or local compression, they seem not to fail at specific locations while the spike is being propagated. This may indicate that branching is usually necessary to stop an already propagating spike.

### What prevented the failures?

Drugs that broadens the spike often prevent failures (Bostock et al. 1978; Westerfield et al. 1978; Swadlow et al. 1980). Our results show that this is the case also in thin, unmyelinated axon in the mammalian cortex.

We used TEA because it is known to broaden the parallel fiber spike (Sabatini and Regehr 1997). However, cerebellar granule cells express more than 26 K^+^ channel *α*‐subunits, including six delayed rectifier types (Mathie et al. 2003), and TEA reduces several of them, but usually at higher concentration than what is needed to block the Kv3 family of channels (Rudy and McBain 2001). Based on pharmacological studies of the grease‐gap recorded parallel fiber cAP (Pekala et al. 2014), we find it likely that the Kv3 family dominates the fast repolarization. In that article, the Kv1‐blocking toxins changed the cAP much less than 1 mmol/L TEA. Only when TEA‐sensitive channels were blocked, Kv1‐blocking increased the cAP significantly. Quinine, that blocks not only Kv2 channels but also several other channels, did not have any detectable effect on the fast cAP, unless both Kv3 and Kv1 channels were blocked. Together, those results suggest that Kv1 channels participated less than Kv3 as long as Kv3 channels were operational, and that the spike broadening was the main mechanism by which 1 mmol/L TEA reduced conduction failures.

Since Kv3‐family channels are among the fastest spike repolarizing channels, it is tempting to suggest that these channels may be limiting for the spike amplitude and width, and therefore cause failures when they become even faster at high temperature. However, the rate of Na^+^ channel inactivation and K^+^ activation will both increase with temperature, and it seems difficult to determine which of those changes are most important for reduction of spike amplitude and width (Yu et al. 2012) without having voltage‐clamp control of the membrane.

### The DAP reduced failures

The DAP has been studied in several preparations and so have conduction failures, but the influence of a DAP on conduction failures has to the best of our knowledge not been studied. The DAP has never been recorded directly, as a membrane potential, in unmyelinated small‐bouton CNS axons, but several lines of evidence, some illustrated by experiments in the present article, strongly support its existence in cerebellar parallel fibers and hippocampal stratum radiatum axons (Soleng et al. 2004; Palani et al. 2012; Pekala et al. 2014). For example, with threshold axonal activation and somatic membrane potentials that allowed antidromic somatic spike invasion, the fast spike and the DAP always occurred or failed concurrently, meaning that the DAP was a part of the spike. At somatic potentials that blocked invasion of the fast spike component, the DAP could still be detected, meaning that the DAP was created in the axon and not by the soma.

The hypothesis that the DAP reduced the failure rate is supported by results from the following experiments:

1. In intrasomatic recordings, the DAP eliminated somatic invasion failures. Since the axon‐to‐soma transition is the largest transition from small to large diameter found in most neurons, introducing a particularly large impedance mismatch, the finding that the DAP eliminated these somatic invasion failures makes it likely that the DAP can reduce failures also further out in the axon.
2. In trains of four stimuli, increasing temperatures reduced the first more than the last cAP. Importantly, this tendency was strongest at the temperature where the two‐electrode recordings showed signs of conduction failures. A likely interpretation is therefore that an excitability‐increasing effect of the preceding spikes made it easier for the following spikes to propagate successfully. The DAP offers an explanation for such spike‐dependent excitability‐increasing effect.
3. The cAP latency was reduced from the first to the second response, but almost identical for response 2, 3, and 4. This is compatible with an excitability‐increasing process of equal magnitude after each spike in the four‐stimulus train. When we showed that the DAP, measured either on antidromic action potentials, or far out in the axon with grease‐gap recordings, have very similar amplitude in such four‐stimulus trains, it is likely that the DAP was the process that facilitated propagation, and helped the axons overcome temperature‐induced conduction failures.

Unfortunately, antidromic spikes detected in somatic recordings were not a suitable approach for demonstrating that the DAP could reduce temperature‐induced failures (we used somatic hyperpolarization to induce invasion failures). Firstly, the intracellular recordings were often not stable at temperatures >38°C, which added to the already difficult task of activating the axon belonging to the cell recorded from. Secondly, there is a theoretical but likely problem that only a narrow temperature range allows both successful activation and conduction failures. Below that temperature range, the axons never fail to propagate action potential, and above that range, they may not be able to initiate and action potential. Therefore, to investigate if the DAP influenced temperature‐induced failures further away from the soma, we had to use population recordings.

Could higher temperature increase stimulus‐dependent K^+^ efflux, and thereby contribute to larger cAPs at the end compared to the beginning of the four‐stimulus trains? That is unlikely because such K^+^‐mediated excitability increase would accumulate during the train, conflicting with the finding that excitability, measured as latency reduction jumped from the first to the second stimulus, but thereafter was almost constant for stimuli 2–4. Furthermore, potassium accumulation is unlikely to be the major excitability‐increasing factor because the failure rate for antidromic invasion at the soma actually increased with number of stimuli for stimuli that were *not* preceded by a successfully propagated spike. This strongly suggests that it was the propagation of a spike, and not the stimulus by itself, that increased the excitability.

The most direct proof that the DAP reduces failure rates would be to compare failure rates with and without the DAP present. Unfortunately, this is currently not possible because there is no known method to reduce the DAP (although it can be enhanced by Kv blockers as described in Pekala et al. 2014).

### DAP is robust

The remarkably constant excitability increase, measured in terms of latency reduction of the responses following the initial one in trains of four stimuli, suggests that the DAP is relatively insensitive to activity. This was supported by somatic recordings of antidromic spikes, and grease‐gap recordings of axonal spike populations. The relatively activity‐independent DAP amplitude is similar to recordings using voltage‐sensitive dye presynaptically in crayfish neuromuscular junction (Lin 2012), but has to the best of our knowledge not been studied before in mammals. Furthermore, the DAP was still present at fever‐like temperatures in axons far away from the soma.

### Function

In conclusion, we have found that typical unmyelinated, small‐bouton CNS axons can fail even after a spike has been successfully initiated. The theoretical but very likely possibility that regions of impedance mismatch, sensitive to geometrical factors, play a role in these failures is important because axonal geometry may change during development and in disease. For example, large axonal swellings occur on both myelinated and unmyelinated axons in neurodegenerative‐relevant mouse models (Sanchez‐Varo et al. 2012).

The relatively moderate temperature increase giving conduction failures makes them potentially important for understanding confusion, fatigue, and seizures occurring at high body temperatures. The tendency of the young animals to fail at a lower temperature than the older ones (Fig. 2D) may contribute to an understanding of the higher seizure susceptibility of young rats and perhaps humans (Dube et al. 2007). However, evolution could obviously have pushed the failure temperature higher, so since these mammalian axons have this failure mechanism at temperatures just above normal body temperature, it may serve a protective function, for example, by limiting the axons' energy expenditure at high temperatures.

## Conflict of Interest

None declared.
